# Perinatal outcome of pregnant mothers with active rubella infection in a tertiary hospital in Nigeria

**DOI:** 10.4314/gmj.v59i1.3

**Published:** 2025-03

**Authors:** Beatrice O Owolabi, Kikelolmo T Adesina, Omotayo O Adesiyun, Abayomi Fadeyi, Adebunmi O Olarinoye, Sherifat T Suleiman, James O Owolabi, Dele O Amadu

**Affiliations:** 1 University of Ilorin Teaching Hospital Ilorin, Department of Obstetrics and Gynaecology; 2 University of Ilorin Teaching Hospital, Department of Paediatrics and Child Health; 3 University of Ilorin Teaching Hospital, Department of Medical Microbiology and Parasitology; 4 University of Ilorin Teaching Hospital, Department of Radiology

**Keywords:** pregnant mothers, congenital infections, rubella virus, Immunoglobulin M, Immunoglobulin G

## Abstract

**Objective:**

To determine the seroprevalence of the rubella virus among pregnant women and the perinatal outcome of neonates of mothers with active rubella virus.

**Design:**

A cross-sectional and longitudinal study

**Setting:**

The study was conducted in the Obstetrics and Gynaecology Department of a teaching hospital in Nigeria.

**Participants:**

Pregnant women at the gestational age of 18-20 weeks.

**Intervention:**

Screening pregnant mothers for immunoglobulin (IgG, IgM) for the rubella virus. Neonates of pregnant mothers with active rubella infection (IgM positive) were screened at birth for rubella IgM to assess for congenital rubella infections and congenital rubella syndrome.

**Main outcome measure:**

Prevalence of rubella immunoglobulin G and active rubella infection IgM, congenital rubella infection, and congenital rubella syndrome.

**Results:**

Of the 327 participants, 68.8% were rubella IgG seropositive, while 7.6% were Rubella IgM seropositive. Fifty-six (56) per cent of neonates of women with active rubella infection were Rubella IgM seropositive at birth, and 14.3% of the neonates of Rubella IgM seropositive pregnant women with active rubella infection had occipitofrontal circumference of less than 10^th^ percentile for gestational age.

**Conclusion:**

Rubella virus is present in our environment with the risk of pregnant mother and neonate of being infected

**Funding:**

None declared

## Introduction

Rubella is a single-stranded RNA (ribonucleic acid) virus, the only member of the genus Rubivirus, and belongs to the Togaviridae family.^1^ It is named German Measles because it was discovered by German physcicians.^2^ Rubella is a teratogenic virus; it is the cause of congenital rubella syndrome and congenital abnormalities when infection occurs in pregnancy.^3^ Congenital infection can lead to miscarriages, fetal death, or the birth of an infant with congenital rubella syndrome. It has been estimated that over 100,000 cases of congenital rubella syndrome occur in developing countries each year.^4^

The prevalence of rubella antibody among pregnant women in Ibadan, Nigeria, was 68.5%; in Benin City, Nigeria, it was 53%; the prevalence in Maiduguri, Northeastern Nigeria, was 54.1%.^5^,^6^,^7^ The seroprevalence of rubella virus antibodies in Bangladesh was 84.3%.^8^ Rubella is transmitted by droplet infection and direct contact with nasopharyngeal secretions of infectious persons. The portal of entry of the rubella virus is the upper respiratory tract. 9 The incubation period is 2 to 3 weeks.^10^ Humans are the only host for rubella. In pregnant women with clinical or inapparent rubella, the virus infects the placenta during the period of viremia and subsequently infects the foetus.

Maternal infection may result in no infection of the conceptus, resorption of the embryo, spontaneous abortion, stillbirth, or infection of the placenta and the fetus.^11^ Gestational age at the time of maternal infection is the most critical determinant of intrauterine transmission and fetal damage.^11^ A rubella IgM positive result in a single serum sample indicates an acute rubella infection or a fourfold rise in immunoglobulin G (IgG) in a serum sample taken sequentially and then assayed in pairs. The detection of Rubella-specific immunoglobulin G (IgG) is indicative of exposure to the rubella virus either from wild-type virus infection or from vaccination.^12^ Congenital rubella infection can be confirmed in the laboratory by detection of Rubella-specific IgM in the serum or cord blood of the infant.^14^ Congenital rubella syndrome is confirmed when the infant has at least one of the following symptoms: cataracts or congenital glaucoma, microcephaly, congenital heart disease, hearing impairment, pigmentary retinopathy, and laboratory evidence of rubella infection demonstrated by either isolation of rubella virus or detection of Rubella specific immunoglobulin(IgM)antibody or a specimen that is polymerase chain reaction positive for Rubella virus.^15^ The development and use of a live attenuated rubella vaccine has controlled rubella virus infection in developed countries during the last 40 years, even enabling elimination in Nordic countries and the region of the Americas.^15^

Nevertheless, congenital rubella remains an essential problem in countries that do not use vaccines against the rubella virus, especially in developing countries such as Nigeria.^15^ Elimination of Rubella and congenital syndrome is now a goal throughout the western hemisphere promoted by the Pan American Health Organization.^16^ Sub-Saharan Africa remains a problem, both for epidemiological and economic reasons. The incidence of congenital rubella syndrome is poorly documented.^17^ The best therapy for congenital rubella syndrome is prevention. All girls should be vaccinated against rubella before entering the childbearing years. The provision of universal infant immunization to decrease circulating virus is another prevention measure. Programs to ensure postpartum immunization of non-immune women before they are discharged from the hospital are another prevention measure. Rubella infection in pregnancy and congenital infection is a vaccine-preventable disease. In Nigeria, no rubella vaccine policy is available for women of childbearing age. There is a need to assess the prevalence of rubella infection among pregnant women and the perinatal outcomes such as congenital rubella syndrome.

This study was undertaken to determine the prevalence of the rubella virus among pregnant women and the perinatal outcomes of mothers with active rubella infection at the University of Ilorin Teaching Hospital Ilorin. The specific objectives were:

1. To determine the seroprevalence of Immunoglobulin G (IgG) to rubella virus among pregnant women.
2. To determine the prevalence of rubella infection (Immunoglobulin M IgM) in pregnant women.
3. To determine the vertical transmission of rubella virus in mothers with active rubella infection to their newborns.
4. To determine the foetal and neonatal outcomes of babies of mothers with active rubella infection.

## Methods

### Setting

The study was conducted in the Department of Obstetrics and Gynaecology, University of Ilorin Teaching Hospital, Kwara State, Nigeria. The hospital is located in Ilorin, the capital of Kwara State. The hospital is situated along old Jebba Road Ilorin, and it serves as a major referral center for all areas in Kwara and parts of the neighbouring state of Kogi, Ekiti, Osun, Oyo, and Niger.

### Study Population

The study population consisted of pregnant women who attended the antenatal clinic and were then admitted to the labour ward of the University of Ilorin Teaching Hospital for delivery.

### Study Design

The study was conducted in two stages. The first stage was a cross-sectional study to determine the prevalence of rubella immunoglobulin IgG and rubella infection IgM (using both rubella-specific immunoglobulin G and M) among pregnant women. The longitudinal components involved following up on the Rubella IgM seropositive pregnant women and screening their newborns at birth to identify the Rubella virus's vertical transmission rate. The sociodemographic characteristics of Rubella IgM seropositive pregnant women were compared with those of Rubella IgM seronegative women. The study was a hospital-based prospective study. Consenting pregnant women in the antenatal clinic who met the criteria were recruited for the study at booking (between gestational ages of 18-20 weeks). Pregnant participants were recruited using a simple random sampling technique. A sample size of 228 was calculated using Fisher's formula.^18^ Participants were counselled about the study, and informed consent was obtained. The study was conducted over 2 years.

The social class of the study participants was determined by the social class classification by Olusanya et al. 19 The classification of socioeconomic status (SES) is divided into upper (1), middle (2), and lower (3) SES.

The scores include the educational attainment of mothers and the occupation of fathers of participants

### Inclusion Criteria

- Pregnant women who presented to the antenatal clinic.
- Booked patient
- Willingness to deliver at UITH Ilorin.
- Consent to participate in the study.

### Exclusion Criteria

- Women with previous vaccination against rubella.

### Sampling Method

**Sampling frame:** A total of 2300 pregnant women booked and delivered in the hospital yearly. Participants were recruited using a simple random sampling technique by balloting, and pregnant women between 18 and 20 weeks were asked to pick from the wrapped papers. A Yes or No paper was wrapped separately and thoroughly mixed. Those who picked yes were recruited, while those who chose No were not recruited for the study.

### Procedures

The participants were counselled about the study, and informed consent was obtained. Pre-test counselling involved risk assessment, assessment of understanding of the disease, and the benefit of testing. A study proforma was administered to each participant. It was designed to obtain their sociodemographic status and other relevant information.

### Collection and Processing of Blood Samples

Five (5) ml of blood sample was obtained by venepuncture after disinfection with 70% alcohol. The sample was collected in a sterile bottle and allowed to clot. The serum was separated in the microbiology laboratory and stored at minus 200 until the time for test analysis.

### Screening of pregnant women for Rubella-specific antibodies

A commercially available enzyme-linked immunosorbent assay (ELISA) kit was used for the qualitative and quantitative determination of immunoglobulin M to Rubella IgM and IgG antibodies in the serum (manufactured by Rapid Laboratory Limited unit 2 Hall farm Church Road Little Bentley Colchester Essex.C07. United Kingdom).

### Procedure of ELISA test For Rubella Specific IgG and IgM

Purified rubella antigen was coated on the surface of the microwells. Diluted patient serum and the Rubella IgM/IgG-specific antibody were added to the wells. If present, it binds to the antigen. All unbound materials are washed away. After adding enzyme conjugate, it binds to the antibody-antigen complex. Excess enzyme conjugate was washed off, and chromogenic substrate was added. The enzyme conjugate catalytic reaction was stopped at a specific time. The intensity of the colour generated is proportional to the amount of IgM/IgG-specific antibodies in the sample. A microwell reader reads the results and compares them parallel with the calibrator and control.

### Interpretation of Rubella Elisa test

A negative result M/G index of less than 0.9 is negative for IgM/IgG antibodies to the rubella virus.

Positive result -rubella M/G index of 1 or greater is positive for IgM antibody to rubella virus.

Interpretation and results.

A positive IgM signifies active rubella infection.

A positive IgG signifies past infection or vaccination.

Screening of neonates

The neonatal cord blood of babies of IgM seropositive mothers with active infection was obtained to detect both IgM and IgG to Rubella using a commercially available enzyme-linked immunosorbent assay (ELISA) kit.

Interpretation of results in neonates

Positive IgM in neonates signifies a congenital infection.

Positive IgG means passive immunization from the maternal antibodies.

### Post-test counselling

After consenting, participants were tested and counselled about the test results. Those with active infection were counselled about the risk of the disease. Those who tested negative, meaning they were not immune to rubella infection, were advised to be vaccinated against rubella after delivery.

### Definitions of terms

1. Low birth weight was less than 2.5kg, while average birth weight was between 2.5-3.9kg.
2. Congenital rubella syndrome was confirmed if there was laboratory detection of Rubella-specific immunoglobulin (IgM) antibody and one of the clinical evidence of microcephaly and or congenital cataracts.
3. OFC less than 10th percentile for gestational age.^20^
4. Active infection: rubella-specific immunoglobulin M(IgM) antibodies.

### Foetal and Perinatal Outcome

The fetal and perinatal outcomes were assessed by determining those that had miscarriages, intrauterine fetal death, stillborn, congenital rubella syndrome, and unaffected neonates. Those outcomes were determined by following up with them from the time of booking to the time of delivery. Neonatal outcome was assessed by determining the first and fifth-minute Apgar score, birth weight of the baby, requirement for NICU admission, indication for NICU admission, duration of admission, and neonatal state at discharge. The neonates' occipitofrontal circumference (OFC) was measured and charted using tape. Lubchenco chart was used for charting OFC.^20^ OFC of less than 10th percentile for gestational age or less than 2 standard deviations for gestational age is microcephaly. The eyes of the neonates were examined with a pen torch to look out for cataracts.

### Sample size determination

The sample size was determined using Fisher's formula, and a sample size of 228 was calculated using a prevalence of 16% of rubella antibodies in Ilorin.^21^ The study's power was 85%, with a confidence level of 95%.

### Ethical Consideration

Approval for the study was obtained from the ethical committee of the University of Ilorin Teaching Hospital with reference UITH/CAT/189/17A/806

### Data analysis

Each participant in the study had one data proforma sheet completed for her. The information from all the data sheets was analyzed using the version of the Statistical Package for Social Science (SPSS) software. The results were expressed as percentages and mean with standard deviation. The continuous variables were analyzed using the student t-test, while categorical variables were analyzed with the chi-square test. A p-value of < 0.05 was taken as significant. Fischer exact test was also used for analysis with small sample or event.

## Results

Three hundred twenty-seven pregnant women were tested for rubella immunoglobulin G and IgM. The mean age of the participants was 29.64±4.64 years. Most of them, 312(95.4%), were married, 4.3% were single, and 1 was a divorcee. All the study participants had more than basic primary education. More study participants, 296(90.5%), were of the Yoruba tribe, while 61.2% were multigravidas. The mean weight was 70.22 Kg, while the mean BMI was 27.91 kg/m^2^. This is shown in Table I

**Table 1:** Socio-demographic and clinical characteristics of pregnant women

Variable	Frequency n(%)
**Age (years)**	
**< 35**	280(85.6)
≥ **35**	47(14.4)
**Marital status**	
**Single**	14(4.3)
**Married**	312(95.4)
**Divorced**	1(0.3)
**Educational level**	
**None**	6(1.8)
**Primary**	19(5.8)
**Secondary**	61(18.7)
**Tertiary**	241(73.7)
**Tribe**	
**Yoruba**	296(90.5)
**Others**	31(9.5)
**Social class**	
**Low**	37(11.3)
**Middle**	168(51.4)
**High**	122(37.3)
**Gravidity**	
**1**	92(28.1)
**2 - 4**	200(61.2)
**> 4**	35(10.7)

Of the 327 participants, (225)68.8% were IgG rubella positive, 31.2% were IgG negative, and (25)7.6% were positive for immunoglobulin IgM. Nine (2.8%) were positive for both Rubella IgG and IgM.

Seven (7.1 %) of the participants were less than 35 years of age among Rubella IgM-positive women, while 92.9% of this age group were Rubella IgM-negative. Ten 10.6% of Rubella IgM positive were more than 35 years while 89.4% of Rubella IgM negative were more than 35 years of age. Most of the Rubella IgM-positive women were in the middle class. The mean weight of those IgM positive was 69.00±8.77 kg, while the mean weight of IgM negative was 58.20±4.71kg. The mean BMI of Rubella IgM positive women was 27.95±3.51Kg/m2 while that of Rubella IgM negative was 23.48±2.06kg. There was a statistically significant difference in weight and body mass index when rubella IgM-positive women were compared with Rubella IgM-negative women (p value=0.018). This is shown in Table 2.

**Table 2:** Association between IgM seroprevalence and demographic variables

Immunoglobulin IgM					
	Positive	Negative	Total	χ^2^	*P*-value
Variable	n(%)	n(%)	N		
**Age**					
**< 35**	20(7.1)	260(92.9)	280	0.696^F^	0.379
≥ **35**	510.6)	42(89.4)	47		
**Marital status**					
**Single**	0(0.0)	14(100.0)	14	1.474^F^	0.641
**Married**	25(8.0)	287(92.0)	312		
**Divorced**	0(0.0)	1(100.0)	1		
**Educational status**					
**None**	0(0.0)	6(100.0)	6	5.200^F^	0.129
**Primary**	0(0.0)	19(100.0)	19		
**Secondary**	9(14.8)	52(85.2)	61		
**Tertiary**	16(6.6)	225(93.4)	241		
**Tribe**					
**Yoruba**	19(6.4)	277(93.6)	296	6.650^F^	0.021*
**Others**	6(19.4)	25(80.6)	31		
**Social class**					
**Low class**	0 (0.0)	37(100.0)	37	5.933	0.051
**Middle class**	18 (10.7)	150(89.3)	168		
**High class**	7 (5.7)	115(94.3)	122		
**Gravidity**					
**1**	8 (8.7)	84(91.3)	92	0.335	0.846
**2 – 4**	15 (7.5)	185(92.5)	200		
**> 4**	2 (5.7)	33(94.3)	35		

**χ^2^: Chi square test; F: Fisher's exact test**

* ***p* value <0.05**

### Neonatal Outcome

Fifty-six 56% of babies of Rubella IgM seropositive mothers were Rubella IgM positive, while 44% were Rubella IgM negative. None of the babies had rubella IgG. Two (14.3%) of Rubella IgM seropositive neonates had an occipitofrontal circumference of less than the 10^th^ percentile for the gestational age. The combination of IgM positivity and microcephaly in the two neonates signifies congenital rubella syndrome. None of the neonates had congenital cataracts at birth.

Obstetric Outcome of Rubella IgM seropositive mothers Of the 25 rubella IgM seropositive pregnant women, the mean duration rupture of the membrane was 3.56±2.63 hours, while the mean duration of labour was 6.44±1.75 hours. Sixty-four 64% of them had caesarean section, while 36% had a vaginal delivery. The mean first-minute APGAR score was 6.76±1.60, while the fifth-minute APGAR score was 8.18±1.24. The mean birth weight was 3.06±0.43kg. The mean occipitofrontal circumference was 35.09±1.95. Only 1 of these 25 babies needed NICU admission. All the babies were discharged alive, as shown in Table III.

**Table 3:** Neonatal outcome of neonates of a mother with Immunoglobulin M (IgM)

Variable	Frequency (n = 25) n(%)
**Mode of delivery**	
**Vaginal delivery**	9(36.0)
**Caesarean section**	16(64.0)
**NICU admission**	
**Yes**	1(4.0)
**No**	24(96.0)
**Neonatal stage at discharge**	
**Alive**	25(100.0)

Indication for admission: Perinatal asphyxia

## Discussion

The prevalence of Rubella IgG was 68.8% in this study. This is comparable to 63.3% in a study done in Kaduna State.^22^ The value obtained was lower than the prevalence of 86.1% of rubella IgG in a study done in Rivers State, Nigeria.^23^ Ninety-two (92.3%) of women screened in Oyo State were positive for Rubella IgG, which was higher than the value obtained in this study.^24^ A prevalence of 64.1% of Rubella IgG was found in a study by Caroca et al in Sao Tome and Principe.^25^ This figure is close to the prevalence found in this study. Greater than 70% of women of childbearing age had rubella antibodies in a study done in Ghana, whereas a low level of 33% was found in Togo.^26^ There is the possibility of geographical variation in the prevalence of the rubella virus. Rubella virus is endemic in Nigeria, which makes women of reproductive age susceptible to infection and reinfection, thereby increasing the risk of congenital rubella infection. Also, the high prevalence of Rubella IgG is presumably from the immunity induced by previous rubella infection. None of the participants was vaccinated against the rubella virus.

Thirty-one (31.2%) of the participants were Rubella IgG negative, implying that they had no previous immunity to the rubella virus. In a study done in Burkina Faso, 5% of pregnant women were seronegative to rubella virus, which was lower than the finding in this study.^27^ In a survey carried out in Morocco, 14.1% of pregnant women were Rubella IgG seronegative, which was also lower than the finding in this study. 28 In Namibia, 15% of pregnant women were seronegative to rubella virus, which is also lower than the finding in this study.^29^ In this endemic environment where there is no vaccination program for the rubella virus, Rubella seronegative women are at risk of being infected and its attending complications like congenital rubella syndrome.

IgM seropositivity in 7.6% of the participants signifies an active rubella infection in pregnancy. The finding in this study is comparable to Rubella IgM prevalence of 5% found in Yaoundé in Cameroon.^30^ In a study done by Praveen RS in India, a prevalence of 5% was found, which was comparable to the findings in this study.^31^ Also, a study carried out in Port-Harcourt, Nigeria, a prevalence of 5.5% of Rubella IgM was found.^32^ In a study carried out in Ethiopia, 39.5% were IgM positive, which was more than the prevalence of this study.^33^ Furthermore, rubella IgM seropositivity of 6.84% was found in women of reproductive age in a study done in India, similar to Rubella IgM seropositivity in this study.^34^

In relating IgM rubella positivity to the sociodemographic variables, it was in keeping with studies done in Kaduna and Cameroon, where rubella IgM was more prevalent in women under 35 years of age group.^22^,^30^. Also, a study done in Saudi Arabia by Alshamlam et al., where younger women were more susceptible than older women, was in keeping with the findings in this study.^35^ Rubella IgM positivity was more common in women who were overweight, those with a mean BMI of 27.95±3.51 Kg/m2. When the BMI of Rubella IgM-positive participants was compared with that of IgM-negative participants, there was a significant difference between the two groups. The finding in this study was in keeping with that of Alshamlam et al, where rubella seropositivity was more common among overweight women. An association between susceptibility to rubella infection and body mass index may exist. Two 2.8% of the pregnant women were seropositive to both IgG and IgM. It can either be due to a new infection or reinfection status of this infection can be determined by rubella IgG avidity.

Fifty 56% of babies born to Rubella IgM-positive mothers were IgM-positive, which implies a congenital Rubella infection in the neonates. This is comparable to the rate of congenital rubella infections in a study by Mirambo et al., where 52.7% of babies born to IgM-positive mothers had congenital rubella infections at birth.^36^ One in 10 of the babies born to women with rubella infection were likely to come down with congenital rubella syndrome in a study done in Tanzania.^36^ Fourteen 14.3% of Rubella IgM-positive neonates born to Rubella IgM-positive mothers had occipitofrontal circumference OFC of less than the 10th percentile for gestational age. The findings of congenital rubella infection (IgM positive) and microcephaly in these neonates imply the presence of congenital rubella syndrome (CRS). In a similar study in Tanzania, 10.9% of mothers with congenital rubella infection had babies with CRS.^36^ There were no miscarriages and stillbirths among the Rubella IgM-positive mothers in this study. Also, none of the fetuses with congenital infection at birth had cataracts.

## Conclusion

Rubella virus is prevalent in our environment, with our pregnant mothers and their neonates at risk of being infected.
